# Respiratory Muscle Training Can Improve Cognition, Lung Function, and Diaphragmatic Thickness Fraction in Male and Non-Obese Patients with Chronic Obstructive Pulmonary Disease: A Prospective Study

**DOI:** 10.3390/jpm12030475

**Published:** 2022-03-16

**Authors:** Yuan-Yang Cheng, Shih-Yi Lin, Chiann-Yi Hsu, Pin-Kuei Fu

**Affiliations:** 1 Department of Physical Medicine and Rehabilitation, Taichung Veterans General Hospital, Taichung 407219, Taiwan; rifampin@gmail.com; 2 Department of Post-Baccalaureate Medicine, College of Medicine, National Chung Hsing University, Taichung 402010, Taiwan; 3 School of Medicine, National Yang Ming Chiao Tung University, Taipei 11221, Taiwan; sylin@vghtc.gov.tw; 4 Center for Geriatrics and Gerontology, Taichung Veterans General Hospital, Taichung 40705, Taiwan; 5 Biostatistics Task Force of Taichung Veterans General Hospital, Taichung 407219, Taiwan; chiann@vghtc.gov.tw; 6 Department of Critical Care Medicine, Taichung Veterans General Hospital, Taichung 407219, Taiwan; 7 Integrated Care Center of Interstitial Lung Disease, Taichung Veterans General Hospital, Taichung 407219, Taiwan; 8 College of Human Science and Social Innovation, Hungkuang University, Taichung 433304, Taiwan

**Keywords:** COPD, respiratory muscle training, cognitive impairment, inspiratory muscle training, expiratory muscle training, FEV1, diaphragmatic thickness fraction

## Abstract

Patients with chronic obstructive pulmonary disease (COPD) are frequently comorbid with mild cognitive impairment (MCI). Whether respiratory muscle training (RMT) is helpful for patients with COPD comorbid MCI remains unclear. Inspiratory muscle training (IMT) with or without expiratory muscle training (EMT) was performed. Patients were randomly assigned to the full training group (EMT + IMT) or the simple training group (IMT only). A total of 49 patients completed the eight-week course of RMT training. RMT significantly improved the maximal inspiratory pressure (MIP), the diaphragmatic thickness fraction and excursion, lung function, scores in the COPD assessment test (CAT), modified Medical Research Council (mMRC) scale scores, and MMSE. The between-group difference in the full training and single training group was not significant. Subgroup analysis classified by the forced expiratory volume in one second (FEV1) level of patients showed no significant differences in MIP, lung function, cognitive function, and walking distance. However, a significant increase in diaphragmatic thickness was found in patients with FEV1 ≥ 30%. We suggest that patients with COPD should start RMT earlier in their disease course to improve physical activity.

## 1. Introduction

Chronic obstructive pulmonary disease (COPD) is characterized by chronic airway inflammation, which causes obstructed airflow from the lungs, resulting in muscle wasting and respiratory failure [1]. Currently, approximately 300 million people world-wide have COPD, contributing to approximately 64 million disability-adjusted life years [2]. In addition to smoking cessation, oxygen therapy, and long-acting bronchodilator therapy, comprehensive pulmonary rehabilitation programs involving aerobic exercise, cough technique education, and respiratory muscle training (RMT) are crucial in the management of COPD [3]. Inspiratory muscle training (IMT), the major method of RMT, can improve inspiratory muscle strength, exercise capacity, quality of life, and dyspnea [4]. Therefore, IMT has been recommended as a part of pulmonary rehabilitation programs for patients with COPD [5,6]. Expiratory muscle training (EMT) can improve vital capacity and peak expiratory flow [7,8], which are also beneficial for cough function. In addition, the improvement in cough function is a vital part of pulmonary rehabilitation; therefore, RMT including IMT and EMT plays a critical role in the management of COPD [9].

Mild cognitive impairment (MCI) is defined based on the following four criteria [10]: (1) a change in cognition reported by the patient, caregiver, or clinician; (2) objective evidence of impairment in one or more cognitive domains, which typically includes memory; (3) preservation of independence in functional abilities; and (4) absence of dementia. Mini-Mental State Examination (MMSE) scores of 23–27 indicate MCI [11]. Many studies have revealed an association between COPD and MCI [12,13,14,15], and a dose–response relationship between the duration of COPD and the risk of MCI [13,14]. Furthermore, forced expiratory volume in one second (FEV1) is positively correlated with cognitive function throughout adulthood [16] because of the higher risk of neuronal injury in patients with chronic hypoxemia [17]. The chronic generalized inflammatory status of patients with COPD can affect MCI pathogenesis [18]. Although aerobic exercise can improve the cognitive function of patients with dementia [19,20,21], only one study has evaluated the effect of IMT on cognitive function [22]. Because FEV1 is associated with cognitive function, and RMT can improve FEV1 performance, RMT may improve cognitive function. However, whether RMT provides additional benefits for the cognitive function of patients with COPD whose MMSE scores are within the range of MCI remains unclear.

Because RMT is an important part of pulmonary rehabilitation, the aim of the current study investigated the improvement of cognition, lung function, clinical scores, and diaphragmatic muscle performance before and after the introduction of RMT in populations of COPD comorbid with mild cognitive impairment. In subgroup analysis, we want to compare the training efficacy between the full training group (EMT + IMT) and the single training group (IMT only) on cognition, lung function test, clinical scores, and diaphragmatic muscle performance. Finally, we will evaluate the RMT efficacy on patients with different severities of lung function impairment classified by the forced expiratory volume in one second (FEV1) level less than 30%.

## 2. Materials and Methods

### 2.1. Participants

Our study was a prospective study that was approved by the Institutional Review Board-I (107-A-09 Board Meeting) of Taichung Veterans General Hospital (protocol code: CF18259A; date of approval: 4 October 2018; clinical trial number: NCT04929990). Participants were recruited from the outpatient department of chest medicine in a tertiary referral center, and written informed consent was obtained from them or their authorized representatives before enrolment. Patients with the following criteria were enrolled: (1) a definitive diagnosis of COPD based on a FEV1/forced vital capacity (FVC) value of less than 0.7 at 10–15 min after short-acting beta-2 agonist (SABA) inhalation, and (2) an MMSE score between 23 and 27. The exclusion criteria were as follows: (1) being unable to follow RMT instructions or complete the questionnaires of our study due to cognitive impairment; (2) difficulty in completing cardiopulmonary exercise testing (CPET) or the 6 min walking test (6MWT) due to high-risk cardiopulmonary diseases or orthopedic conditions, such as critical aortic stenosis, early stage of post myocardial infarction, or lower limb amputation; (3) a diagnosis of lung cancer or a history of thoracoabdominal surgery; and (4) a body mass index (BMI) of ≥30.

### 2.2. Protocol of Intervention

After signing the informed consent form, the participants were assigned to the full RMT training group (EMT + IMT) or the single RMT training group (IMT only) through simple randomization (i.e., tossing a coin). Neither the participants nor the examiner were blinded. The patients enrolled into the current study performed both full RMT training or single RMT training using a threshold-type breathing trainer (Dofin DT11/14, Galemed, Taipei, Taiwan) at the hospital, and the RMT program included 30 breaths two times a day, 5 days a week, for a total of 8 weeks at home. IMT was performed after complete and slow air expiration, followed by quick and forceful air inspiration to overcome the threshold resistance of the device. By contrast, EMT was performed after complete and slow air inspiration, followed by quick and forceful air expiration. In the full RMT training group (IMT + EMT), before training, maximal inspiratory pressure (MIP) and maximal expiratory pressure (MEP) were measured using a digital pressure gauge (GB60, Jitto International, Taipei, Taiwan), and the best performance of the three trials was recorded. The procedure of MIP/MEP measurement resembled that of IMT/EMT training, except the breathing trainer was replaced with the digital pressure gauge. The initial intensity of training was set at 30% of MIP and MEP. The intensity was adjusted with the addition of 5% resistance each week, and a well-trained assistant contacted the participants telephonically to remind them to adjust the intensity every week.

In the simple RMT training group, only IMT was performed using the same type of Dofin DT11/14 breathing trainer. The initial resistance of the breathing trainer was set at 30% of MIP, and subsequent adjustments were made in accordance with the protocol of the experimental group. The participants were instructed to perform training for 30 breaths twice daily for 8 weeks at home, and they were also telephonically supervised by the same assistant every week.

### 2.3. Parameter Measures

Parameter measures in the current study were MMSE score; diaphragmatic thickness fraction and excursion examined through ultrasound; scores of the COPD assessment test (CAT) and modified Medical Research Council (mMRC) scale; percentage of predicted FVC, FEV1, FEV1/FVC, diffusing capacity of the lung for carbon monoxide (DLCO), and DLCO divided by alveolar volume (VA) examined using a pulmonary function test; dead space fraction (Vd/Vt) and minute ventilation to CO_2_ output (VE/VCO_2_) slope examined using the cardiopulmonary exercise test (CPET); and distance walked and changes in oxygen saturation (SpO_2_) and perceived exertion (Borg scale) during six-minute walking test (6MWT). All these measures were assessed the day before the initiation of the RMT program and again 8 weeks later at the end of the program.

**Figure 1:**
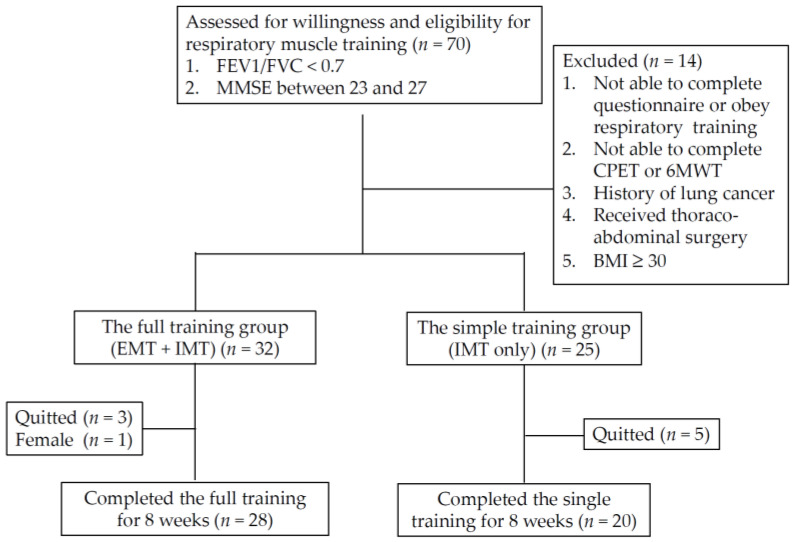
Participant recruitment flowchart: BMI: body mass index; CPET: cardiopulmonary exercise test; IMT: inspiratory muscle training; EMT: expiratory muscle training; 6MWT: six-minute walking test.

Diaphragmatic thickness fraction and excursion were measured using an ultrasound machine (Alpinion E-cube i7, who Medical Co., Ltd., Taipei, Taiwan). With the participant in the supine position, diaphragmatic thickness at the intercostal space between the 7th and 8th or the 8th and 9th ribs in the anterior axillary line was examined using a high-frequency ultrasound probe (10–15 MHz). The thickness of the diaphragmatic apposition zone was visualized below the intercostal muscles (Figure 1). The diaphragmatic thickness fraction was calculated as follows: (end-inspiration thickness—end-expiration thickness)/end-expiration thickness [23]. Diaphragmatic excursion was measured by placing a 2–6-MHz ultrasound probe at the right mid-clavicular line, and the amount of movement of the posterior edge of the liver was traced and measured using M mode ultra-sonography [24]. Limitations, such as the variations of probe tilting angle and the impact of the increased echogenicity of the liver, were considered in our study. The sonographic measurements were performed by one single examiner, which could reduce the inter-observer variability, and none of our participants’ liver echogenicity was too high to clearly identify the posterior edge of liver.

CAT consists of eight questions, each scored from 0 to 5. mMRC only has one question, which is graded from 0 to 4. Both questionnaires are useful in discerning the respiratory difficulty encountered in daily life for patients with COPD, and in categorizing them for guiding treatment [25]. Regarding the assessment of dementia severity, the MMSE is one of the most widely adopted questionnaires in health care settings. The highest total score on the MMSE is 30, and a score of 23–27 indicates MCI [11], which was used in our study.

For patients with COPD, the pulmonary function test is crucial for grading disease severity, and predicting prognosis. In patients with FEV1/FVC < 0.7 [26], COPD severity can be further categorized into four groups according to the extent to which FEV1 reaches the predicted level: <30%, 30–50%, 50–80%, and >80%. In addition to spirometry data, a diffusion study, including DLCO and DLCO/VA, was conducted using a pulmonary function measurement machine (Vmax Encore VS229, Carefusion Co., Ltd., San Diego, CA, USA).

In patients with COPD, exercise performance and cardiopulmonary endurance frequently worsen as the disease progresses. Moreover, 6MWT involves walking as far as possible for 6 min, and is a common indicator of oxidative capacity in patients with cardiopulmonary diseases [27]. We established 6MWT distance by having our participants walk back and forth on a 30 m-long walkway with two cones placed at both ends, and we recorded changes in oxygen saturation and perceived exertion on the Borg scale during 6MWT. In addition, a cardiopulmonary exercise test can detect any gas exchange abnormalities during exercise in patients with COPD [28,29]. Patients with COPD have a higher VE/VCO_2_ slope [29] and Vd/Vt [28] during exercise. In this study, we measured these parameters during peak exercise. CPET was performed using an electro-magnetically braked cycle ergometer, and a mask was used to collect the partial pressure of O_2_ and CO_2_ simultaneously; a 10 W/min ramp protocol was used. All the testing procedures followed the guidelines of the American Heart Association [30].

### 2.4. Statistical Analysis

SPSS 17.0 (IBM, Chicago, IL, USA) was used to perform the statistical analysis. Categorical variables were presented as frequency and percent, and analyzed using the chi-squared test to determine significance. For nonparametric data distribution, differences between groups were assessed using the nonparametric Mann–Whitney U test or Wilcoxon signed ranks test. Results are presented as the mean and standard deviation (SD). To determine the sample size, we adopted G*Power 3.1.9.7 (Heinrich-Heine-Universität Düsseldorf, Germany) to analyze improvements in the MMSE score after physical exercise training, as described previously [21]. With a difference in the improvement of MMSE between 2.67 ± 1.88 and 0.2 ± 2.87 under the setting of α = 0.05 and power –1 − β) = 0.8, the effect size was 1.018, and at least 36 cases were deemed necessary to achieve sufficient statistical power. All tests were two-sided, with *p* < 0.05 considered significant.

## 3. Results

### 3.1. Patients’ Clinical and Demographic Characteristics

From June 2019 to February 2021, a total of 70 patients were enrolled into the study, and 49 participants completed the 8-week course of the RMT program for the final analysis. Because there was only one female patient, the study also excluded her data to reduce the effect of gender difference. Table 1 presents a summary of the demographic characteristics, and clinical and physiological parameters of all participants. Among them, 29 and 20 participants were included in the full RMT training group (IMT + EMT) and the single training group (IMT only), respectively. Figure 1 presents the flowchart of participant recruitment and the case numbers in the subgroup of RMT training. Classified by the FEV1 level, 28.6% of participants were <30% (*n* = 14), and 71.4% of patients were ≥30% (*n* = 35).

### 3.2. Differences between before and after RMT Program

Before and after RMT were compared with respect to all parameters (Table 2). After RMT, patients exhibited significant improvements in FEV1 (%), CAT score, mMRC score, MMSE score, MIP (cmH_2_O), MEP (cmH_2_O), SpO_2_ at rest (%), diaphragmatic thickness fraction, and diaphragmatic excursion (all *p* < 0.01). In addition, the Borg scale after 6MWT was significantly decreased after the RMT program (*p* = 0.016). No significant differences were observed in DLCO (%), 6MWT distance (m), and CPET test after RMT program (all *p* > 0.05).

### 3.3. Differences between the Full RMT Training and Single RMT Training Group

**Figure 2:**
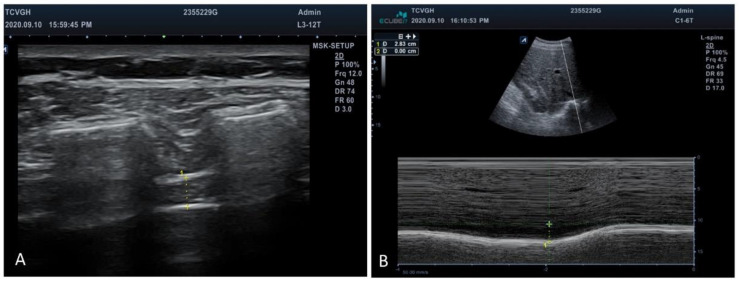
Ultrasonographic evaluation of diaphragm. (**A**) Diaphragmatic thickness was measured below the intercostal muscles between the ribs. +: Markers of the anterior and posterior edges of diaphragm. (**B**) Amount of diaphragmatic excursion was measured using the M mode to trace the movement of the posterior edge of liver. +: Markers of the posterior edge of liver during respiration.

The characteristics of the full RMT training and single RMT training groups were compared (Table 3). After 8 weeks, both groups exhibited increases in lung function, MIP, diaphragmatic excursion, and thickness fraction (Figure 2). However, the between-group difference in the full training (IMT + EMT) and single training (IMT only) groups was not significant in all parameters listed in Table 3.

### 3.4. Differences of RMT Training Effect between FEV1 < 30% and FEV1 ≥ 30% among Patients with COPD

**Figure 3:**
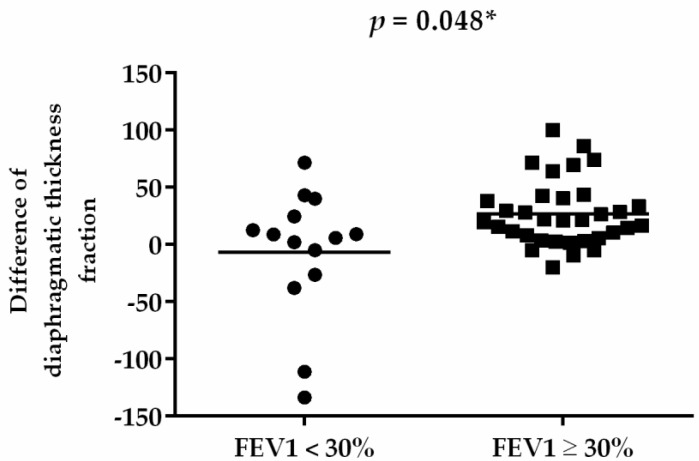
Difference of diaphragmatic thickness fraction before and after RMT. * *p* < 0.05.

The subgroup analysis of the RMT training effect between different severities of FEV1 in patients with COPD was compared (Table 4). After 8 weeks, both groups exhibited increases in FVC (%), FEV1 (%), and the distance of 6MWT; and decreases in CAT score, mMRC score, and Borg scale sore (Table 4). In addition, cognitive function in terms of the MMSE score improved in both groups. However, only diaphragmatic thickness fraction exhibited significant between-group differences in improvement (*p* = 0.044) (Figure 3).

## 4. Discussion

This study yielded three major findings. First, the results of our study indicated that RMT, both in the full training group (IMT + EMT) and simple training group (only IMT), could significantly improve not only cognition, but also inspiratory strength, diaphragmatic performance, FEV1, and dyspnea scores in the patients with COPD comorbid with mild cognitive impairment. Second, we observed that even with a baseline FEV1 of <30%, benefits could be derived from RMT in terms of the outcome measures. Third, we found that patients with preserved lung function (FEV1 ≥ 30%) significantly increased in diaphragmatic thickness fraction after RMT training. The strength of current study is that it is the first to use not only clinical score and lung function test, but also apply both diaphragmatic ultrasonography and the cardiopulmonary exercise test to evaluate the effect of RMT. To the best of our knowledge, this is the first study to evaluate the different RMT training models and the impact of RMT in different severities of patients with COPD.

IMT was necessary in both full and simple RMT training groups in the current study, and the FEV1 was improved after RMT training. Several studies have demonstrated that IMT can elevate FEV1 [31,32,33,34] due to the improvement in trunk control, with more favorable respiratory biomechanics [35]. In addition, IMT can improve cognitive function, as reported in a previous study [22]. Many studies have demonstrated the relationship between hypoxemia and cognitive impairment [36,37]; however, we cannot conclude that the cognition improvement of our patients was due to the increase of resting SpO_2_. That is because the level of SpO_2_ in our participants never reached the threshold level of hypoxemia. Since FEV1 level was positively correlated with cognitive function [16], we suggest that the cognition improvement of this cohort was due to the increase in FEV1 rather than the increase of resting SpO_2_ after RMT implementation. However, the underlying mechanism requires further study.

Our study revealed that RMT implementation, both in the simple and full training group, improved not only cognition, and FEV1 and SpO_2_ at rest, but also MIP, diaphragmatic thickness fraction, diaphragmatic excursion, and CAT and mMRC scores. The results are consistent with those of previous studies [9,38,39]. Weiner et al. reported that dyspnea was alleviated in IMT-only and IMT + EMT groups, but not in the EMT-only group [38]. Weiner and McConnell concluded that no additional benefit was obtained by adding EMT to IMT [39]. Xu et al. revealed improved scores of mMRC, CAT, and St George’s Respiratory Questionnaire in both IMT and IMT + EMT groups, with no significant between-group differences [9]. One important reason is that expiration is predominantly accomplished by elastic recoil instead of active muscle contraction, and therefore, the improvement of inspiratory capacity can also facilitate the performance of expiration. On the contrary, the improvement of expiratory strength may not be so practical in daily respiration, and thus, the indicators of life quality, such as CAT and mMRC scores, cannot be further improved through EMT. Our results also showed that the between-group difference in the full training (IMT + EMT) and single training (IMT only) groups was not significant in all parameters. In addition, our study further provided the image evidence of ultrasonography in diaphragmatic excursion and thickness fraction to support the viewpoint that IMT is the most important part of RMT.

In the current study, the VE/VCO_2_ slope and Vd/Vt during peak exercise did not significantly change after the training program in either group. The VE/VCO_2_ slope, also known as exercise ventilatory efficiency, is an essential prognostic factor in COPD. Vd/Vt, also called the dead space fraction, is considered a comprehensive marker of gas exchange in patients with COPD [40]. RMT cannot enhance ventilator efficiency, and the dead space fraction is probably because RMT can only improve respiratory muscle strength, and not alveolar function of gas exchange.

Most of the previous studies recruited patients with higher FEV1, as easy fatigue during training and poor compliance of training protocol are more common in patients with FEV1 < 30% [41,42,43]. However, the subgroup analysis of the current study revealed no significant between-group difference exhibited in patients with FEV1 < 30% and ≥30%. A study compared the effect of IMT between patients with baseline FEV1 < 50% and ≥50%, and revealed that patients with poor lung function (FEV1 < 50%) demonstrated significant improvement in the sensation of dyspnea after 3 weeks of respiratory training [44]. In our study, we found that the decreases of CAT score and mMRC score were larger in patients with FEV1 < 30%, although it did not reach the statistical difference, which may owe to the small case numbers in the FEV1 < 30% group. A recent meta-analysis demonstrated the benefit of IMT in improving COPD parameters; the authors reported that a shorter intervention time (≤4 weeks) improved MIP only, and a longer training period (6–8 weeks) also improved functional capacity, such as 6MWT distance [45]. Although the training period of our study was up to 8 weeks, no significant change was found in the 6MWT distance. One primary reason for this may be that our participants’ MMSE scores were in the range of MCI, which may affect their ability to achieve full exertion during 6MWT. On the other hand, 6MWT distance reflects cardiopulmonary aerobic capacity, which could be improved only after aerobic exercise training theoretically. RMT, which is a form of strength training, can produce little effect on aerobic capacity. Therefore, inconsistent results were also mentioned in the past meta-analysis regarding the effect of RMT on 6MWT distance [45]. Further studies may be required to elucidate it.

A major strength of our study is that we incorporated the result of diaphragmatic ultrasonography to validate improvements in MIP and MEP. Furthermore, parameters including the VE/VCO_2_ slope and Vd/Vt obtained in CPET were analyzed to determine the reason for the change. However, our study has some limitations. First, although the total number of participants was as per the required sample size for this study, only 14 patients had a baseline FEV1 < 30%, which may have been too few to achieve sufficient statistical power. The data of the improvement of diaphragmatic thickness fraction from two patients with FEV1 < 30% even became outliers. The data of the improvement of diaphragmatic thickness fraction from two patients with FEV1 < 30% even became outliers. Future studies should include more patients with COPD and a baseline FEV1 < 30% to validate our findings. Second, MMSE has limited sensitivity and specificity for diagnosing MCI against healthy controls [46]. Our participants only had MCI according to MMSE scores, but they did not have a confirmed diagnosis of MCI. Third, our participants had MMSE scores suggestive of MCI, which may have affected their compliance with the RMT program at home, despite us having assigned an assistant to contact and encourage the participants every week via telephone. Finally, we excluded those with BMI ≥ 30 to improve the reliability of ultrasonographic results, and none of our participants were female. Therefore, our results may only be applicable to male and non-obese patients with COPD.

## 5. Conclusions

Our study results revealed that the 8-week RMT program improved not only cognitive function, but also CAT score, mMRC score, and diaphragmatic thickness in male and non-obese patients with COPD comorbid mild cognitive impairment. In addition, we found that IMT is the most important part of RMT, as the combination of EMT with IMT was not superior to IMT alone. Furthermore, even patients with a baseline FEV1 of <30% derive benefits from RMT. Patients with preserved lung function (FEV1 ≥ 30%) significantly increased in diaphragmatic thickness fraction after RMT training. We suggest that patients with COPD should start to receive IMT earlier in their disease course to increase their respiratory strength, and thus, achieve a high quality of life.

## Figures and Tables

**Table 1:** Demographic characteristics, and clinical and physiological parameters in patients with COPD enrolled into respiratory muscle training program (*n* = 48).

Characteristics	Mean ± SD (*n*, %)
Age (years)	67.23 ± 7.32
Body mass index (kg/m^2^)	23.02 ± 3.89
Pulmonary function test	
FVC (%)	78.77 ± 21.36
FEV1 (%)	41.08 ± 15.26
FEV1/FVC (%)	42.83 ± 14.19
DLCO (%)	78.05 ± 24.63
DLCO/VA	83.76 ± 25.82
Clinical score	
CAT score	14.17 ± 8.39
mMRC score	1.63 ± 0.98
MMSE	24.39 ± 2.50
MIP (cmH_2_O)	69.41 ± 28.02
MEP (cmH_2_O)	85.30 ± 18.07
6MWT	
6MWT distance (m)	328.25 ± 71.72
SpO_2_ at rest (%)	95.19 ± 4.98
Nadir SpO_2_ in 6MWT	92.69 ± 7.97
Borg scale at rest	1.17 ± 1.32
Borg scale after 6MWT	2.89 ± 1.83
Sonography evaluation	
Diaphragmatic thickness fraction	39.38 ± 28.50
Diaphragmatic excursion (cm)	3.00 ± 1.10
CPET	
Vd/Vt	32.80 ± 7.04
VE/VCO_2_ slope	36.81 ± 6.34
FEV1 subgroup	
<30%	14 (29.17%)
≥30%	34 (70.83%)
Respiratory training subgroup	
IMT	20 (41.67%)
IMT + EMT	28 (58.33%)

IMT, inspiratory muscle training; EMT, expiratory muscle training; CAT, chronic obstructive pulmonary disease assessment test; mMRC, modified Medical Research Council; 6MWT, 6-min walking test; Vd/Vt, dead space fraction; VE/VCO_2_, minute ventilation to CO_2_ output; FVC, forced vital capacity; FEV1, forced expiratory volume in 1 s; DLCO, diffusing capacity of the lung for carbon monoxide; VA, alveolar volume; MMSE, Mini-Mental State Examination.

**Table 2:** Comparison of the difference of parameters between before and after RMT program implementation.

Characteristics	before RMT	after RMT	*p* Value
Pulmonary function test			
FVC (%)	78.25 ± 20.68	81.93 ± 19.14	0.318
FEV1 (%)	40.05 ± 15.09	43.75 ± 15.72	0.002 **
FEV1/FVC (%)	42.05 ± 14.27	41.81 ± 15.82	0.372
DLCO (%)	79.83 ± 26.93	80.33 ± 22.62	0.969
DLCO/VA	86.42 ± 31.44	84.67 ± 27.47	0.563
Clinical score			
CAT score	14.17 ± 8.39	9.06 ± 6.06	<0.001 **
mMRC score	1.63 ± 0.98	1.13 ± 0.67	<0.001 **
MMSE	24.39 ± 2.50	26.00 ± 4.13	0.002 **
MIP (cmH_2_O)	64.08 ± 30.42	80.79 ± 36.93	0.001 **
MEP (cmH_2_O)	80.86 ± 23.48	99.81 ± 34.57	0.036 *
6MWT			
6MWT distance (m)	331.28 ± 70.05	338.80 ± 68.91	0.381
SpO_2_ at rest (%)	95.19 ± 4.98	96.67 ± 2.77	0.005 *
Nadir SpO_2_ in 6MWT	92.69 ± 7.97	93.78 ± 4.11	0.466
SpO_2_ change in 6MWT	2.50 ± 5.82	2.89 ± 3.34	0.174
Borg scale at rest	1.17 ± 1.32	0.83 ± 0.97	0.167
Borg scale after 6MWT	2.89 ± 1.83	2.19 ± 1.65	0.020 *
Borg scale change in 6MWT	1.72 ± 1.61	1.36 ± 1.22	0.232
Sonography evaluation			
Diaphragmatic thickness fraction	39.38 ± 28.50	56.40 ± 28.16	<0.001 **
Diaphragmatic excursion	3.00 ± 1.10	3.83 ± 1.31	<0.001 **
CPET			
Vd/Vt	32.83 ± 7.18	32.98 ± 6.54	0.576
VE/VCO_2_ slope	36.63 ± 6.41	36.51 ± 5.62	1.000

CAT, chronic obstructive pulmonary disease assessment test; DLCO, diffusing capacity of the lung for carbon monoxide; EMT, expiratory muscle training; FVC, forced vital capacity; FEV1, forced expiratory volume in 1 s; IMT, inspiratory muscle training; MIP: maximal inspiratory pressure; MEP: maximal expiratory pressure; mMRC, modified Medical Research Council; MMSE, Mini-Mental State Examination; 6MWT, 6-min walking test; SpO_2_, oxygen saturation; Vd/Vt, dead space fraction; VE/VCO_2_, minute ventilation to CO_2_ output; VA, alveolar volume; * *p* < 0.05, ** *p* < 0.01.

**Table 3:** The differences between the single training group (IMT only) and the full training group (IMT + EMT) in patients with COPD after RMT program implementation.

	IMT Only (*n* = 20)	IMT + EMT (*n* = 28)	*p* Value
Pulmonary function test			
FVC (%)	83.11 ± 19.54	80.86 ± 19.20	0.722
FEV1 (%)	46.32 ± 16.93	41.43 ± 14.56	0.371
FEV1/FVC (%)	43.55 ± 18.88	40.24 ± 12.72	0.386
DLCO (%)	80.00 ± 27.95	72.29 ± 15.13	0.624
DLCO/VA	90.00 ± 28.66	83.36 ± 25.68	0.711
Clinical score			
CAT score	7.75 ± 4.46	10.00 ± 6.91	0.396
mMRC score	1.20 ± 0.62	1.07 ± 0.72	0.667
MMSE	25.78 ± 5.47	26.14 ± 3.23	0.585
MIP (cmH_2_O)	75.25 ± 38.30	83.82 ± 37.69	0.714
MEP (cmH_2_O)		99.81 ± 34.57	---
6MWT			
6MWT distance (m)	321.75 ± 73.09	351.92 ± 63.83	0.166
SpO_2_ at rest (%)	95.95 ± 2.67	96.16 ± 3.45	0.289
Nadir SpO_2_ in 6MWT	92.25 ± 4.89	92.72 ± 4.93	0.680
SpO_2_ change in 6MWT	3.70 ± 3.15	3.44 ± 3.88	0.549
Borg scale at rest	0.95 ± 0.89	0.64 ± 0.95	0.160
Borg scale after 6MWT	2.30 ± 1.98	2.60 ± 1.80	0.523
Borg scale change in 6MWT	1.35 ± 1.63	1.96 ± 1.43	0.076
Sonography evaluation			
Diaphragmatic thickness fraction	50.74 ± 28.74	60.44 ± 27.55	0.098
Diaphragmatic excursion (cm)	4.00 ± 1.17	3.72 ± 1.40	0.523
CPET			
Vd/Vt	31.70 ± 8.52	33.96 ± 4.40	0.230
VE/VCO_2_ slope	36.21 ± 5.75	36.75 ± 5.62	0.991

Mann–Whitney U test.

**Table 4:** Comparison of the RMT training effect between FEV1 < 30% and FEV1 ≥ 30% in patients with COPD.

	FEV1 < 30%	FEV1 ≥ 30%	*p* Value
Pulmonary function test			
ΔFVC (%)	3.33 ± 17.41	3.82 ± 18.62	0.821
ΔFEV1 (%)	3.00 ± 5.22	4.00 ± 7.32	0.666
ΔFEV1/FVC (%)	−1.80 ± 13.23	0.43 ± 7.34	0.867
ΔDLCO (%)	9.33 ± 5.03	−2.44 ± 20.28	0.195
ΔDLCO/VA	0.00 ± 6.08	−2.33 ± 10.99	0.864
Clinical score			
ΔCAT score	−6.86 ± 7.49	−4.38 ± 6.92	0.265
ΔmMRC score	−0.86 ± 1.10	−0.35 ± 0.69	0.074
ΔMMSE	2.00 ± 1.87	1.50 ± 2.48	1.000
ΔMIP (cmH2O)	8.33 ± 15.64	15.79 ± 23.35	0.549
ΔMEP (cmH2O)	18.80 ± 6.08	19.00 ± 23.27	1.000
6MWT			
Δ6MWT distance (m)	15.92 ± 65.42	4.21 ± 29.50	0.951
ΔSpO_2_ at rest (%)	1.86 ± 1.21	1.38 ± 4.03	0.152
ΔNadir SpO_2_ in 6MWT	1.57 ± 2.70	0.97 ± 7.20	0.302
ΔBorg scale at rest	0.29 ± 0.76	−0.48 ± 1.43	0.122
ΔBorg scale after 6MWT	−0.71 ± 1.70	−0.69 ± 1.71	0.922
Sonography evaluation			
ΔDiaphragmatic thickness fraction	−6.84 ± 56.35	26.84 ± 28.55	0.048 *
ΔDiaphragmatic excursion (cm)	1.09 ± 1.50	0.73 ± 1.14	0.734
CPET			
ΔVd/Vt	0.46 ± 5.92	0.02 ± 4.94	0.565
ΔVE/VCO_2_ slope	−0.38 ± 3.71	−0.01 ± 5.18	0.678

Mann–Whitney U test. * *p* < 0.05, Δ: value of parameter after training—value of parameter before training.

## Data Availability

The data presented in this study are available on request from the corresponding author. The data are not publicly available due to the regulation of the Institutional Review Board of Taichung Veterans General Hospital in Taiwan.

## References

[1] Rabe K.F., Watz H. (2017). Chronic obstructive pulmonary disease. Lancet.

[2] Ruvuna L., Sood A. (2020). Epidemiology of Chronic Obstructive Pulmonary Disease. Clin. Chest Med..

[3] McCarthy B., Casey D., Devane D., Murphy K., Murphy E., Lacasse Y. (2015). Pulmonary rehabilitation for chronic obstructive pulmonary disease. Cochrane Database Syst. Rev..

[4] Beaumont M., Forget P., Couturaud F., Reychler G. (2018). Effects of inspiratory muscle training in COPD patients: A systematic review and meta-analysis. Clin. Respir. J..

[5] Hill K., Cecins N.M., Eastwood P.R., Jenkins S.C. (2010). Inspiratory muscle training for patients with chronic obstructive pulmonary disease: A practical guide for clinicians. Arch. Phys. Med. Rehabil..

[6] Frangogiannis N.G., Dewald O., Xia Y., Ren G., Haudek S., Leucker T., Kraemer D., Taffet G., Rollins B.J., Entman M.L. (2007). Critical role of monocyte chemoattractant protein-1/CC chemokine ligand 2 in the pathogenesis of ischemic cardiomyopathy. Circulation.

[7] Chigira Y., Miyazaki I., Izumi M., Oda T. (2018). Effects of expiratory muscle training on the frail elderly’s respiratory function. J. Phys. Ther. Sci..

[8] Kim J., Davenport P., Sapienza C. (2009). Effect of expiratory muscle strength training on elderly cough function. Arch. Gerontol. Geriatr..

[9] Xu W., Li R., Guan L., Wang K., Hu Y., Xu L., Zhou L., Chen R., Chen X. (2018). Combination of inspiratory and expiratory muscle training in same respiratory cycle versus different cycles in COPD patients: A randomized trial. Respir. Res..

[10] Albert M.S., DeKosky S.T., Dickson D., Dubois B., Feldman H.H., Fox N.C., Gamst A., Holtzman D.M., Jagust W.J., Petersen R.C. (2011). The diagnosis of mild cognitive impairment due to Alzheimer’s disease: Recommendations from the National Institute on Aging-Alzheimer’s Association workgroups on diagnostic guidelines for Alzheimer’s disease. Alzheimers Dement..

[11] Zaudig M. (1992). A new systematic method of measurement and diagnosis of “mild cognitive impairment” and dementia according to ICD-10 and DSM-III-R criteria. Int. Psychogeriatr..

[12] Kakkera K., Padala K.P., Kodali M., Padala P.R. (2018). Association of chronic obstructive pulmonary disease with mild cognitive impairment and dementia. Curr. Opin. Pulm. Med..

[13] Singh B., Mielke M.M., Parsaik A.K., Cha R.H., Roberts R.O., Scanlon P.D., Geda Y.E., Christianson T.J., Pankratz V.S., Petersen R.C. (2014). A prospective study of chronic obstructive pulmonary disease and the risk for mild cognitive impairment. JAMA Neurol..

[14] Singh B., Parsaik A.K., Mielke M.M., Roberts R.O., Scanlon P.D., Geda Y.E., Pankratz V.S., Christianson T., Yawn B.P., Petersen R.C. (2013). Chronic obstructive pulmonary disease and association with mild cognitive impairment: The Mayo Clinic Study of Aging. Mayo Clin. Proc..

[15] Ranzini L., Schiavi M., Pierobon A., Granata N., Giardini A. (2020). From Mild Cognitive Impairment (MCI) to Dementia in Chronic Obstructive Pulmonary Disease. Implications for Clinical Practice and Disease Management: A Mini-Review. Front. Psychol..

[16] Anstey K.J., Windsor T.D., Jorm A.F., Christensen H., Rodgers B. (2004). Association of pulmonary function with cognitive performance in early, middle and late adulthood. Gerontology.

[17] de la Torre J.C. (1999). Critical threshold cerebral hypoperfusion causes Alzheimer’s disease?. Acta Neuropathol..

[18] Koyama A., O’Brien J., Weuve J., Blacker D., Metti A.L., Yaffe K. (2013). The role of peripheral inflammatory markers in dementia and Alzheimer’s disease: A meta-analysis. J. Gerontol. A Biol. Sci. Med. Sci..

[19] Venturelli M., Scarsini R., Schena F. (2011). Six-month walking program changes cognitive and ADL performance in patients with Alzheimer. Am. J. Alzheimers Dis. Other Demen..

[20] Vreugdenhil A., Cannell J., Davies A., Razay G. (2012). A community-based exercise programme to improve functional ability in people with Alzheimer’s disease: A randomized controlled trial. Scand. J. Caring Sci..

[21] Van de Winckel A., Feys H., De Weerdt W., Dom R. (2004). Cognitive and behavioural effects of music-based exercises in patients with dementia. Clin. Rehabil..

[22] Ferreira L., Tanaka K., Santos-Galduroz R.F., Galduroz J.C. (2015). Respiratory training as strategy to prevent cognitive decline in aging: A randomized controlled trial. Clin. Interv. Aging.

[23] Sarwal A., Walker F.O., Cartwright M.S. (2013). Neuromuscular ultrasound for evaluation of the diaphragm. Muscle Nerve.

[24] Epelman M., Navarro O.M., Daneman A., Miller S.F. (2005). M-mode sonography of diaphragmatic motion: Description of technique and experience in 278 pediatric patients. Pediatr. Radiol..

[25] Halpin D.M.G., Criner G.J., Papi A., Singh D., Anzueto A., Martinez F.J., Agusti A.A., Vogelmeier C.F. (2021). Global Initiative for the Diagnosis, Management, and Prevention of Chronic Obstructive Lung Disease. The 2020 GOLD Science Committee Report on COVID-19 and Chronic Obstructive Pulmonary Disease. Am. J. Respir. Crit. Care Med..

[26] Burkhardt R., Pankow W. (2014). The diagnosis of chronic obstructive pulmonary disease. Dtsch. Arztebl. Int..

[27] Rasekaba T., Lee A.L., Naughton M.T., Williams T.J., Holland A.E. (2009). The six-minute walk test: A useful metric for the cardiopulmonary patient. Intern. Med. J..

[28] Chuang M.L. (2020). Combining Dynamic Hyperinflation with Dead Space Volume during Maximal Exercise in Patients with Chronic Obstructive Pulmonary Disease. J. Clin. Med..

[29] Boutou A.K., Zafeiridis A., Pitsiou G., Dipla K., Kioumis I., Stanopoulos I. (2020). Cardiopulmonary exercise testing in chronic obstructive pulmonary disease: An update on its clinical value and applications. Clin. Physiol. Funct. Imaging.

[30] Guazzi M., Arena R., Halle M., Piepoli M.F., Myers J., Lavie C.J. (2016). 2016 Focused Update: Clinical Recommendations for Cardiopulmonary Exercise Testing Data Assessment in Specific Patient Populations. Circulation.

[31] Weiner P., Man A., Weiner M., Rabner M., Waizman J., Magadle R., Zamir D., Greiff Y. (1997). The effect of incentive spirometry and inspiratory muscle training on pulmonary function after lung resection. J. Thorac. Cardiovasc. Surg..

[32] Abodonya A.M., Abdelbasset W.K., Awad E.A., Elalfy I.E., Salem H.A., Elsayed S.H. (2021). Inspiratory muscle training for recovered COVID-19 patients after weaning from mechanical ventilation: A pilot control clinical study. Medicine.

[33] El-Deen H.A.B., Alanazi F.S., Ahmed K.T. (2018). Effects of inspiratory muscle training on pulmonary functions and muscle strength in sedentary hemodialysis patients. J. Phys. Ther. Sci..

[34] Bostanci O., Mayda H., Yilmaz C., Kabadayi M., Yilmaz A.K., Ozdal M. (2019). Inspiratory muscle training improves pulmonary functions and respiratory muscle strength in healthy male smokers. Respir. Physiol. Neurobiol..

[35] Aydogan Arslan S., Ugurlu K., Sakizli Erdal E., Keskin E.D., Demirguc A. (2022). Effects of Inspiratory Muscle Training on Respiratory Muscle Strength, Trunk Control, Balance and Functional Capacity in Stroke Patients: A single-blinded randomized controlled study. Top. Stroke Rehabil..

[36] Findley L.J., Barth J.T., Powers D.C., Wilhoit S.C., Boyd D.G., Suratt P.M. (1986). Cognitive impairment in patients with obstructive sleep apnea and associated hypoxemia. Chest.

[37] Areza-Fegyveres R., Kairalla R.A., Carvalho C.R.R., Nitrini R. (2010). Cognition and chronic hypoxia in pulmonary diseases. Dement. Neuropsychol..

[38] Weiner P., Magadle R., Beckerman M., Weiner M., Berar-Yanay N. (2003). Comparison of specific expiratory, inspiratory, and combined muscle training programs in COPD. Chest.

[39] Weiner P., McConnell A. (2005). Respiratory muscle training in chronic obstructive pulmonary disease: Inspiratory, expiratory, or both?. Curr. Opin. Pulm. Med..

[40] Chuang M.L., Hsieh B.Y., Lin I.F. (2021). Resting Dead Space Fraction as Related to Clinical Characteristics, Lung Function, and Gas Exchange in Male Patients with Chronic Obstructive Pulmonary Disease. Int. J. Gen. Med..

[41] Garcia S., Rocha M., Pinto P., Lopes A.M., Bárbara C. (2008). Inspiratory muscle training in COPD patients. Rev. Port. Pneumol..

[42] Petrovic M., Reiter M., Zipko H., Pohl W., Wanke T. (2012). Effects of inspiratory muscle training on dynamic hyperinflation in patients with COPD. Int. J. Chron. Obstruct. Pulmon. Dis..

[43] Hill K., Jenkins S.C., Philippe D.L., Cecins N., Shepherd K.L., Green D.J., Hillman D.R., Eastwood P.R. (2006). High-intensity inspiratory muscle training in COPD. Eur. Respir. J..

[44] Beaumont M., Mialon P., Le Ber-Moy C., Lochon C., Peran L., Pichon R., Gut-Gobert C., Leroyer C., Morelot-Panzini C., Couturaud F. (2015). Inspiratory muscle training during pulmonary rehabilitation in chronic obstructive pulmonary disease: A randomized trial. Chron. Respir. Dis..

[45] Figueiredo R.I.N., Azambuja A.M., Cureau F.V., Sbruzzi G. (2020). Inspiratory Muscle Training in COPD. Respir. Care.

[46] Mitchell A.J. (2009). A meta-analysis of the accuracy of the mini-mental state examination in the detection of dementia and mild cognitive impairment. J. Psychiatr. Res..

